# Supplementary figures and images for: Assessment of Red Sea temperatures in CMIP5 models for present and future climate
Source: PLoS One. 2021 Jul 30;16(7):e0255505. doi: 10.1371/journal.pone.0255505 (PMC8323894; doi:10.1371/journal.pone.0255505)

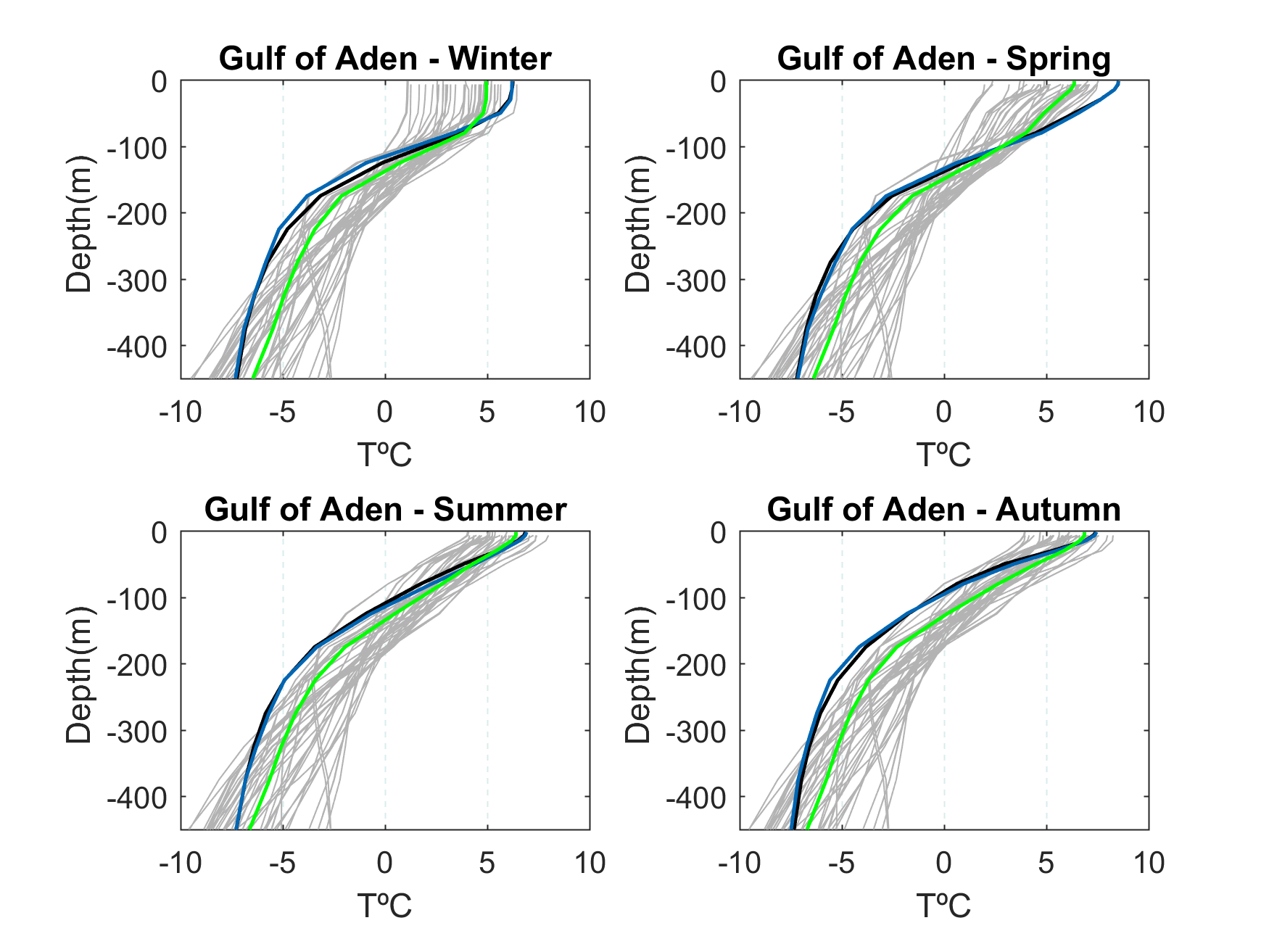

Supplement: S1 Fig — KAUST simulation does not cover this area and is therefore not included in the plots. (TIF) [file pone.0255505.s001.tif]

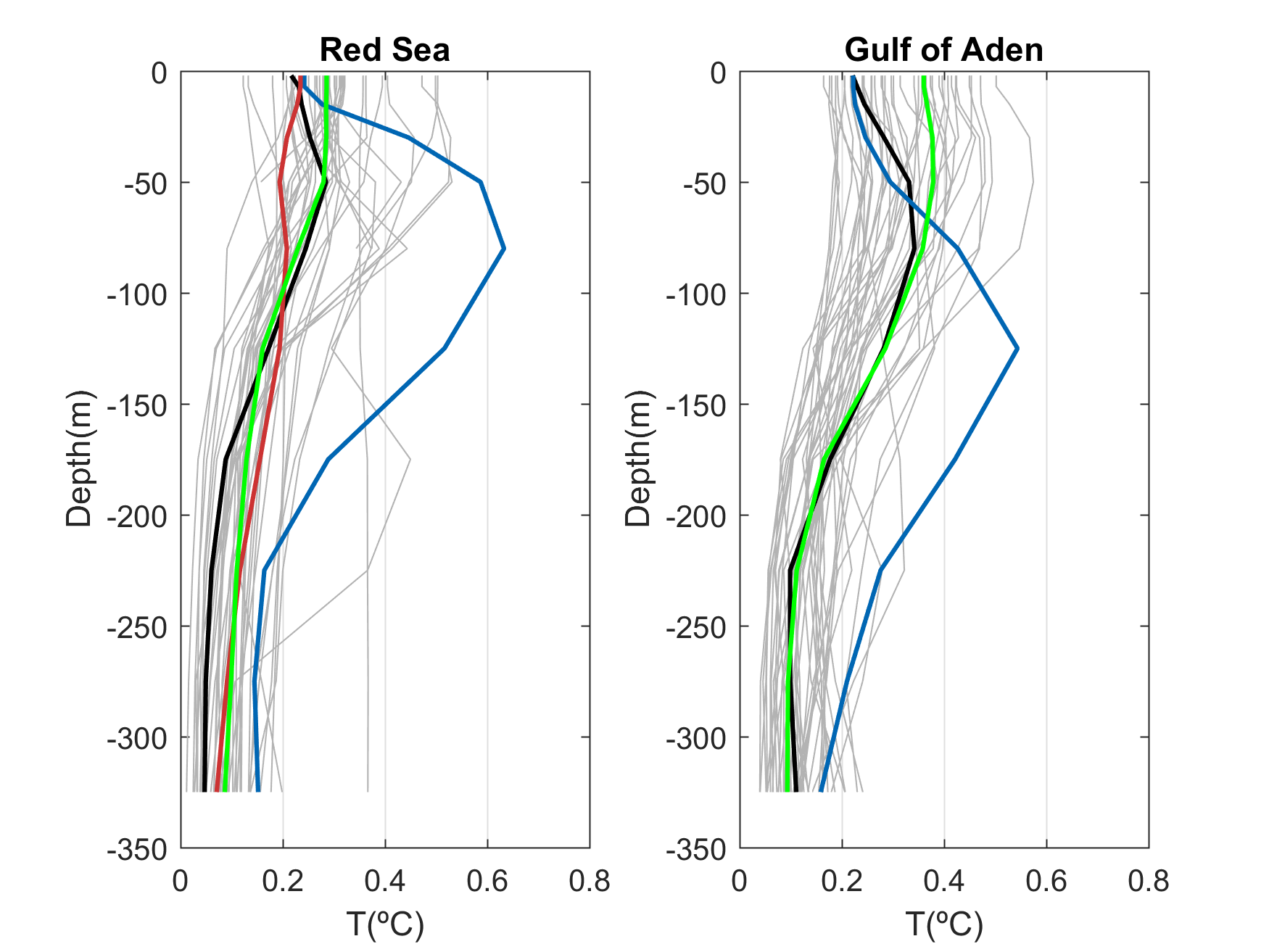

Supplement: S2 Fig — Vertical profile of the interannual STD (in °C) in the Red Sea (left) and in the Gulf of Aden (right) for observations (black line), KAUST (red line), GLORYS (blue line), CMIP5 models (grey lines) and the average of selected GCMs (see “Model Selection” section, green line). (TIF) [file pone.0255505.s002.tif]

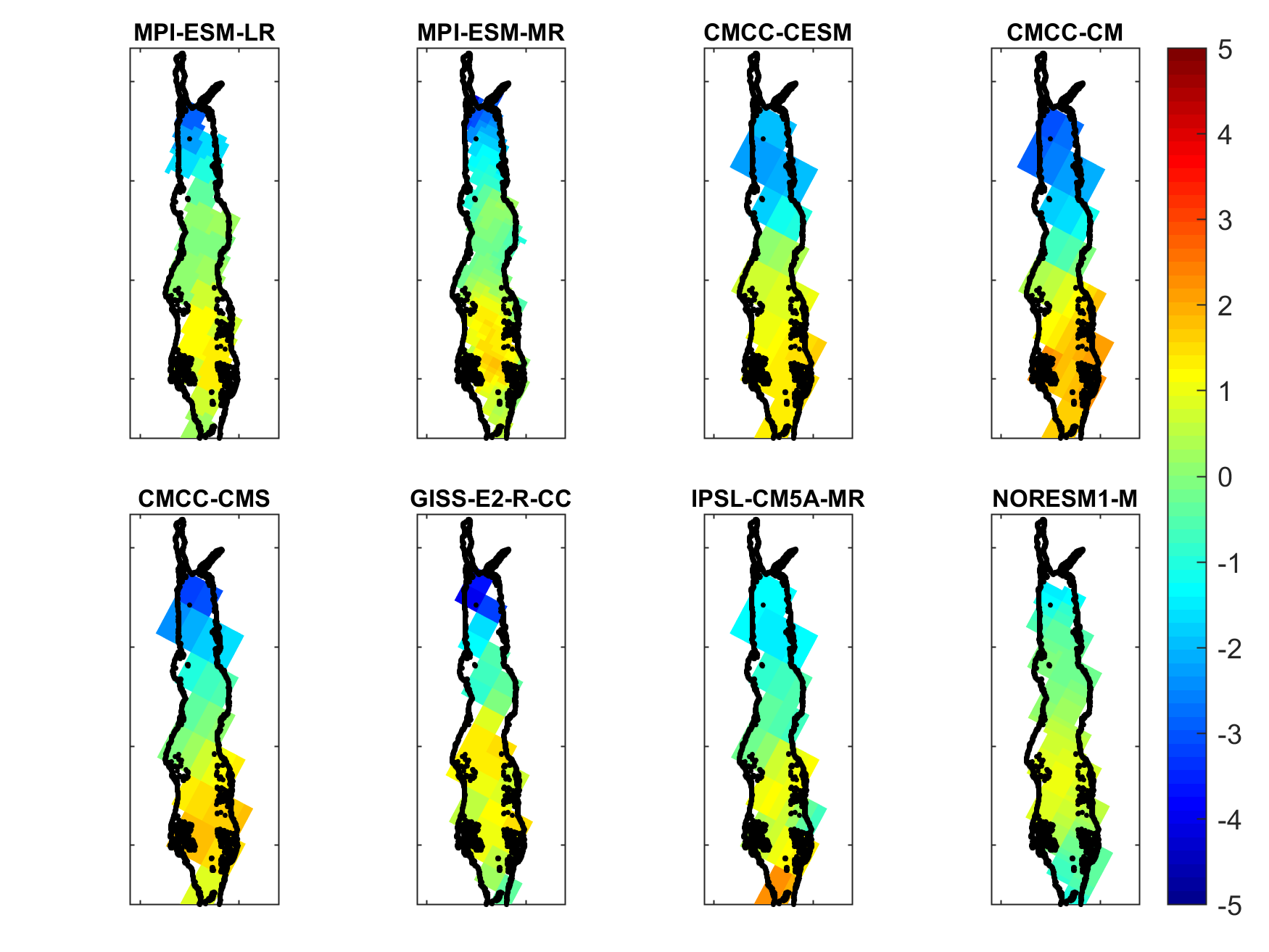

Supplement: S3 Fig — (TIF) [file pone.0255505.s003.tif]

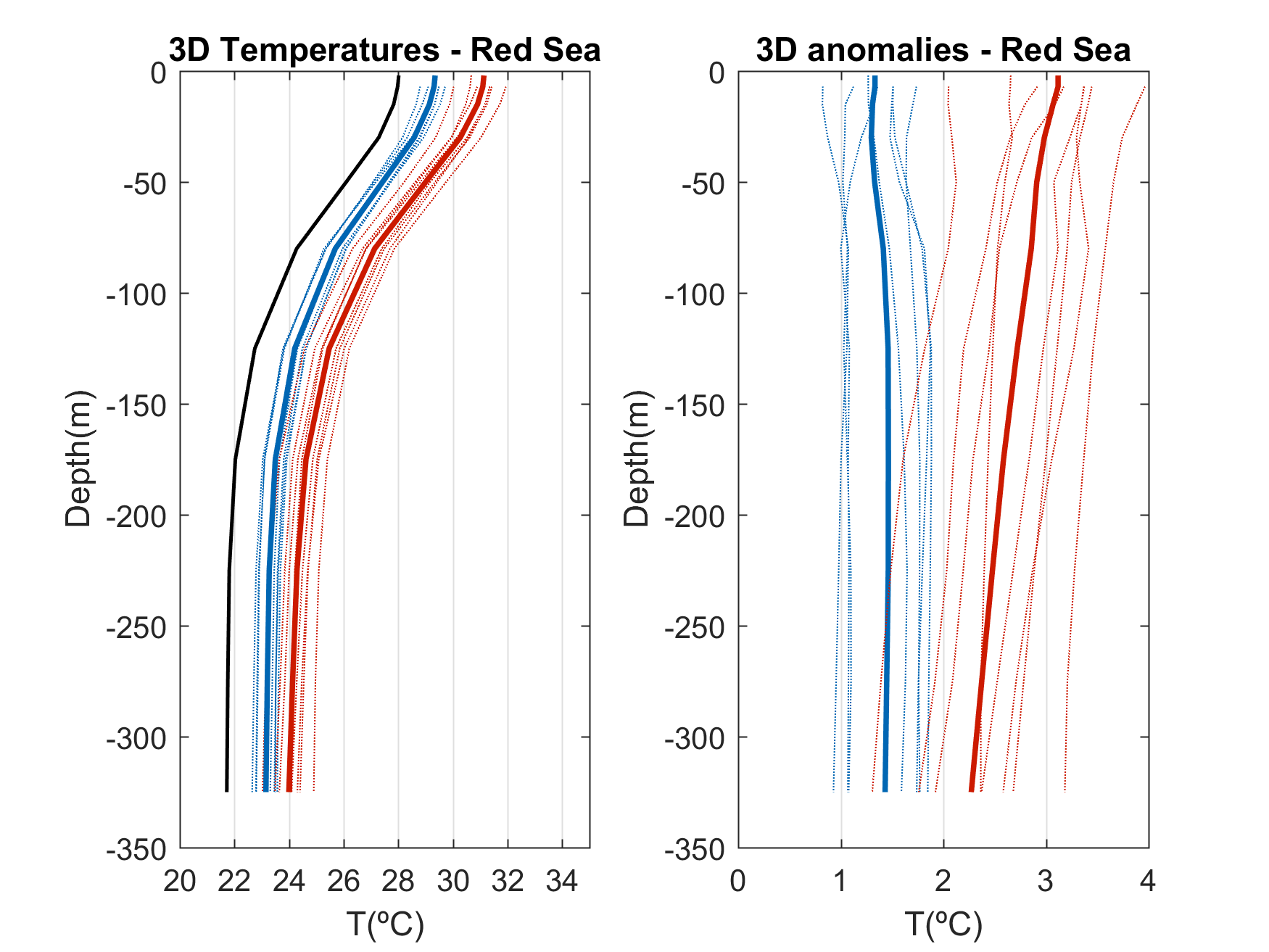

Supplement: S4 Fig — Left panel: Vertical profile of averaged temperatures in the Red Sea (in °C) for the observations (black), RCP4.5 (blue dashed lines, all models selected) and RCP8.5 (red dashed lines, all models selected) and the ensemble average is represented with a thick line. Right panel: Vertical profile of projected anomalies in the Red Sea for all the selected models under scenario RCP4.5 (blue) and RCP8.5(red). The ensemble average is represented with a thick line. (TIF) [file pone.0255505.s004.tif]

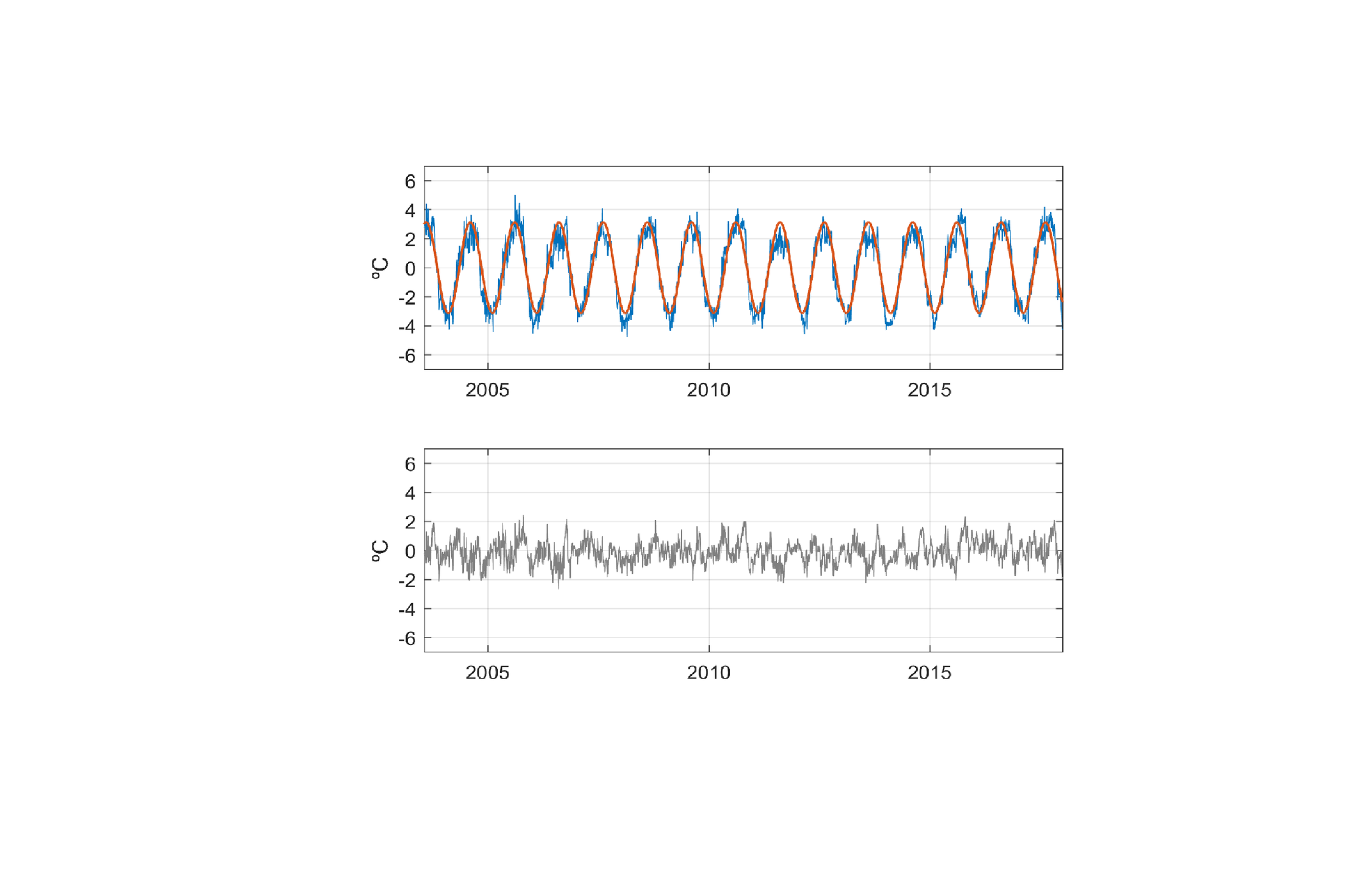

Supplement: S5 Fig — (Top) Anomaly of the observed Sea Surface temperature (blue) and fitted seasonal cycle (red). (Bottom) Intra-seasonal variability of the observed sea surface temperature (grey). (TIF) [file pone.0255505.s005.tif]

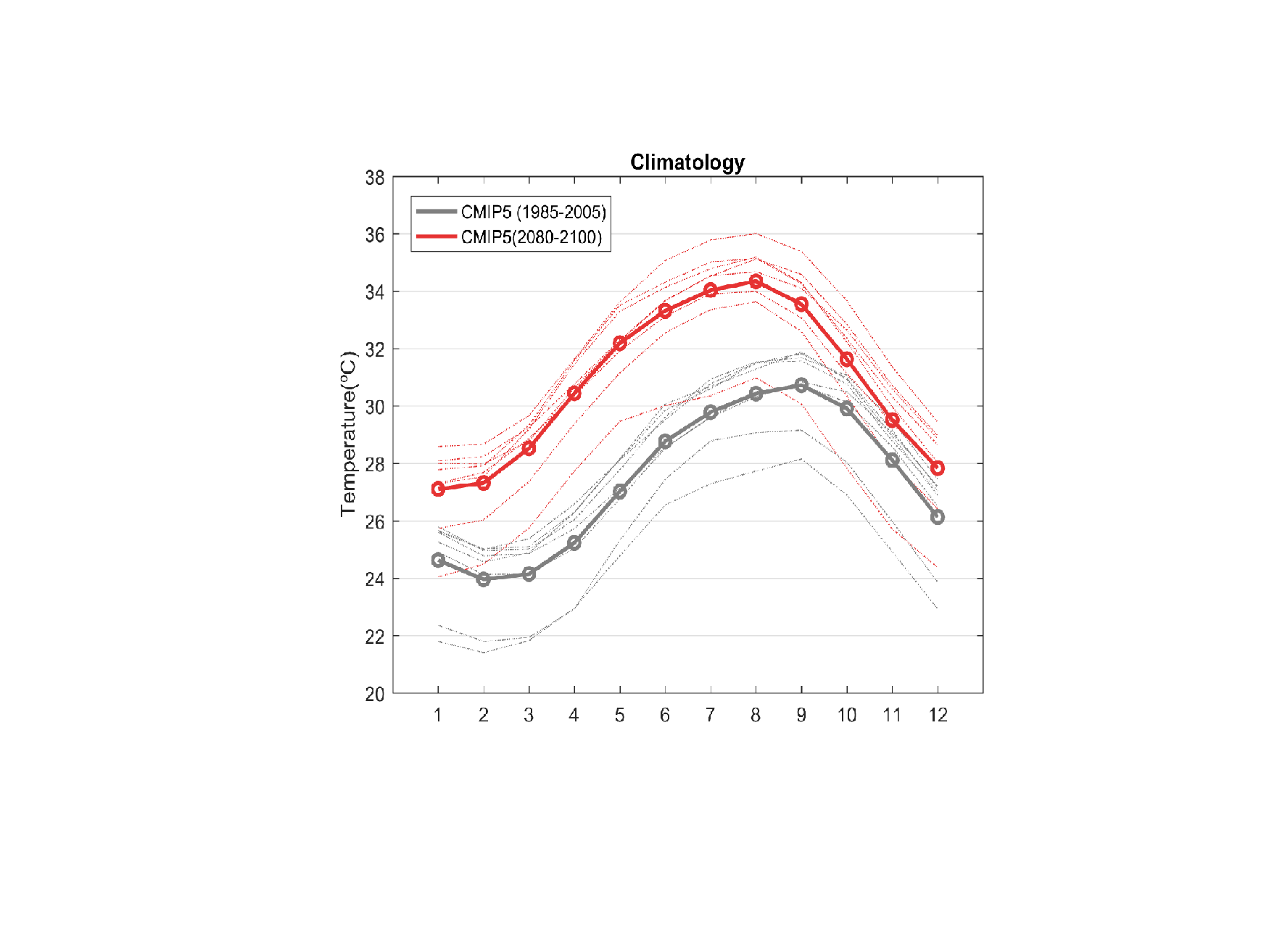

Supplement: S6 Fig — Seasonal climatology of the selected CMIP5 models for Present (grey lines) and Future climate (red lines). The values of each model are represented with thin lines, while the ensemble average is represented with thick lines. (TIF) [file pone.0255505.s006.tif]

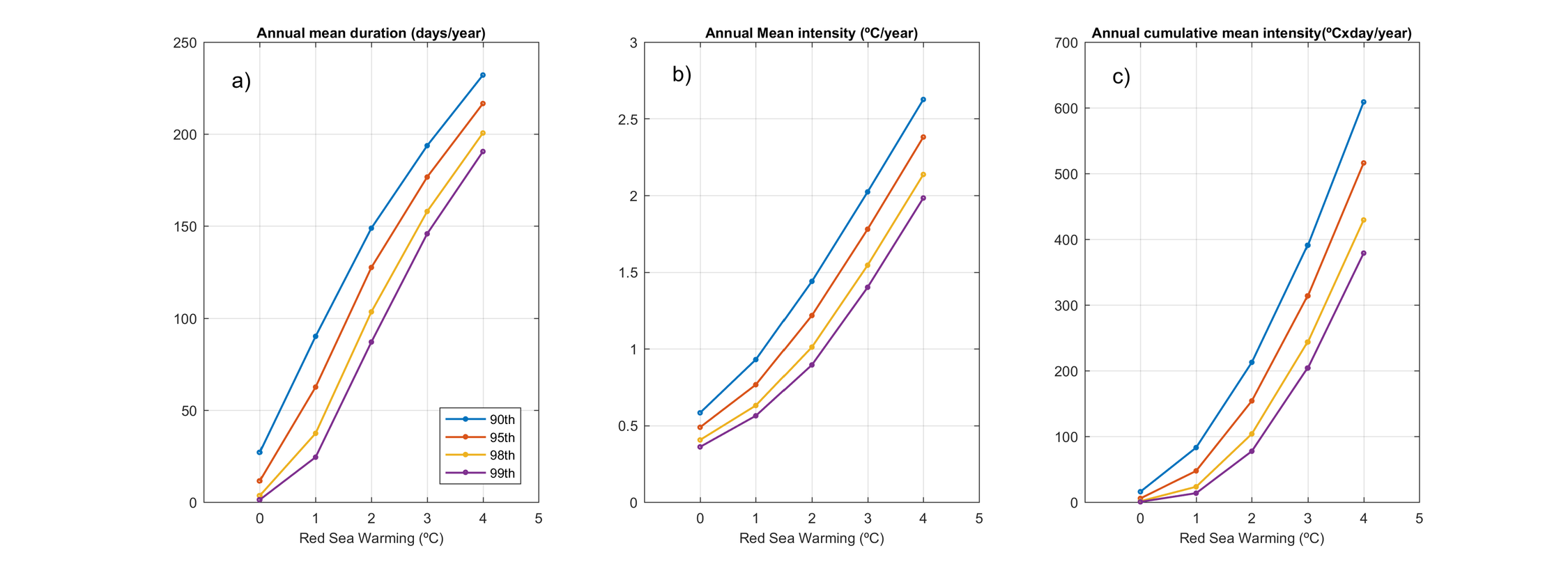

Supplement: S7 Fig — (a) annual mean duration, (b) annual mean intensity and (c) annual cumulative mean intensity (d). The colours represent different thresholds that have been used to define a MHW (90th, 95th, 98th and 99th percentiles). (TIF) [file pone.0255505.s007.tif]

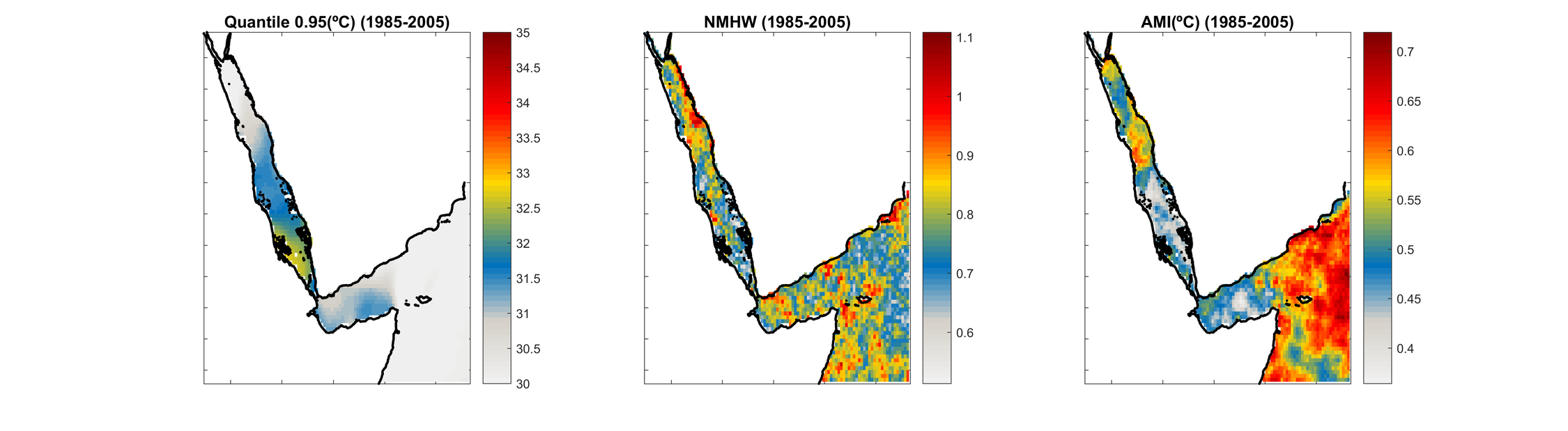

Supplement: S8 Fig — 95th -Percentile (left panel) in °C, number of MHWs per year (middle panel), and the annual mean intensity (AMI) in °C (right panel). (TIF) [file pone.0255505.s008.tif]

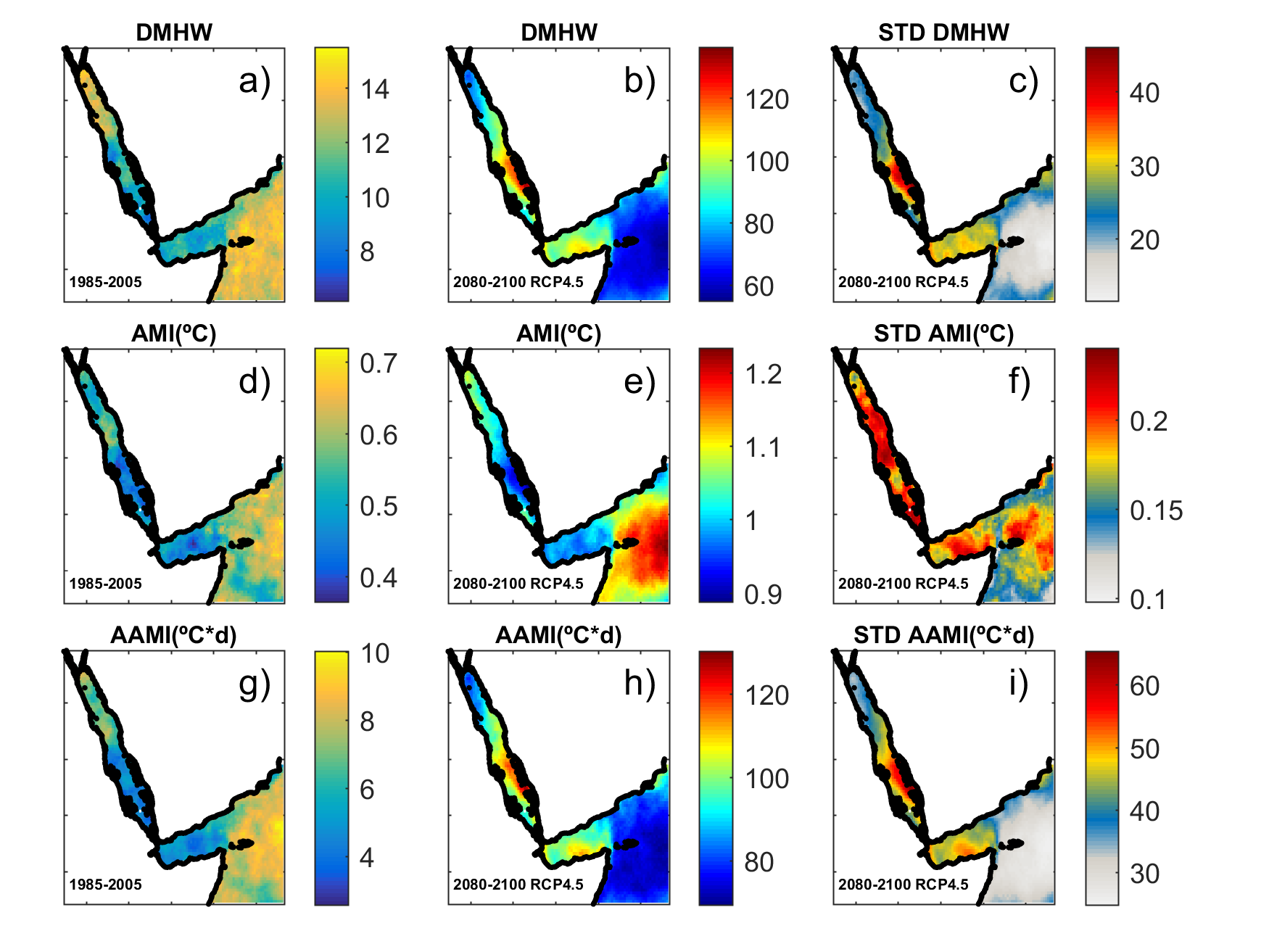

Supplement: S9 Fig — Statistics of MHWs for present conditions (left column), scenario RCP4.5 (ensemble average, middle column) and associated uncertainties (ensemble STD, right column). (a-c) Average length of MHWs in days per year. (d-f) Annual mean Intensity (°C). (g-i) Annual Accumulative mean Intensity (°C · day). Note the colorbars for the future scenarios are different than for the present conditions. (TIF) [file pone.0255505.s009.tif]

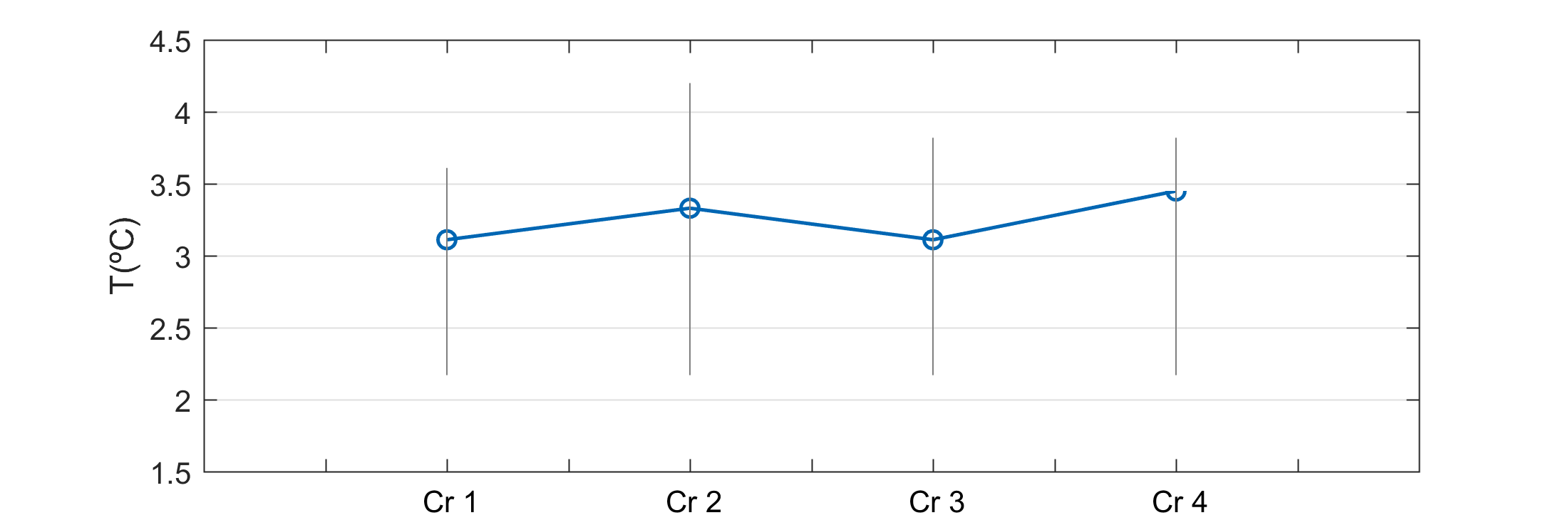

Supplement: S10 Fig — (TIF) [file pone.0255505.s010.tif]

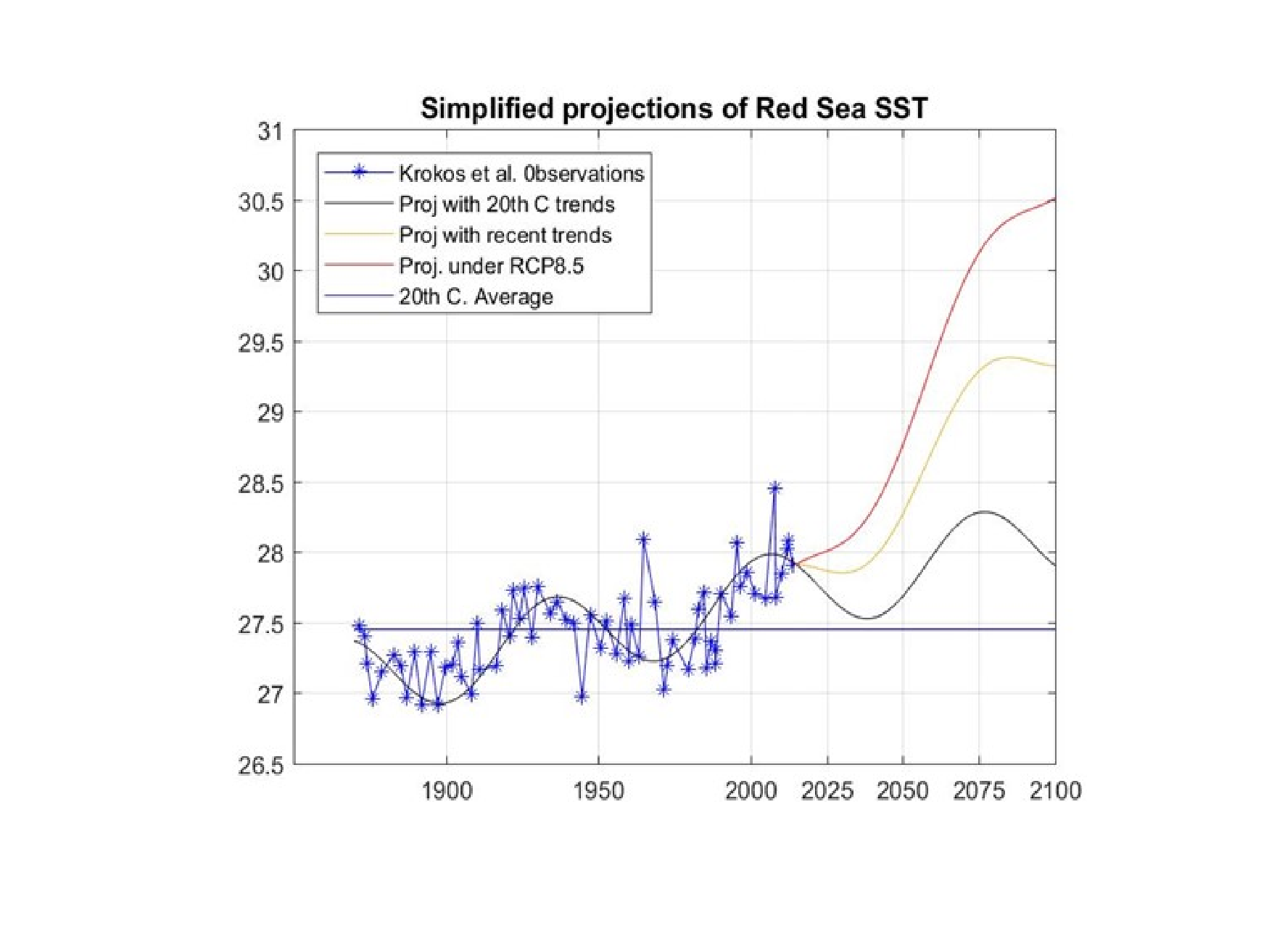

Supplement: S11 Fig — Assuming the multidecadal oscillation identified by Krokos et al., (2019) continues along the 21st century, we test how the temperatures would change imposing different long term trends: The observed 20st century trend (in black), the trend observed during the satellite period (yellow) or the trend projected by models under scenario RCP8.5 (red). (TIF) [file pone.0255505.s011.tif]
