# Supplementary material for: Assessment of Red Sea temperatures in CMIP5 models for present and future climate
Source: PLoS One. 2021 Jul 30;16(7):e0255505. doi: 10.1371/journal.pone.0255505 (PMC8323894; doi:10.1371/journal.pone.0255505)
Supplement: Attachment — Submitted filename: Revision.docx [file pone.0255505.s016.docx]

Revision of “Assessment of Red Sea temperatures in CMIP5 models for present and future climate”

By Agulles M and collaborators.

**General comment**

The work aims to assess the future projection of the Red Sea temperatures in the global warming scenarios of the CMIP5 project. This topic is very relevant and has been poorly studied before. In this sense, I recommend publishing this work in order to increase our knowledge of the GCMs skill to represent the mean state and variability of the temperature field in the Red Sea, as well as the possible consequences that global warming can have in this region. However, there are major comments that should be addressed before publication.

**Major comments**

My major concerns are related to the Marine Heat Waves (MHW) methodology. The methodology used in the paper mixes the information from observations and GCMs in a way that is not usually done. I suggest using the information from the historical experiment to evaluate the GCM skills in representing the main characteristics of MHW respect to observations. As far as I know, there is daily data available for CMIP5 outputs necessary to this purpose (SSTs or air temperature at 2m in the worst case). After this, you can use the present intra-seasonal variability of each model to create the future projections. However, it is very important to keep the individual model projections in order to analyze the multi-model spread and uncertainty. In this part of your results, only the multi model mean is evaluated but in this kind of studies it is very important to discuss the differences that exist between models as a measure of uncertainty.

**Specific comments**

I feel that the document is a little disorganized. Most of the figures do not have proper titles or the units of the color palettes, what makes difficult to read them.

L47. What do you mean by “slow changes”? Do you mean changes at “long term” or changes in “low frequency variability”? Please rewrite this sentence.

L60. There are some very interesting works about global warming and the Red Sea (e.g. “Ocean warming slows coral growth in the central Red Sea” Cantin 2010). For this reason, I suggest changing the phrase “These projections are, however, currently lacking” for something like “The assessment of these projections is limited to few works” and I also recommend citing these few works here.

L123. Please include the units of the color palette (bathymetry).

L154. I didn’t find this information in Table1. Do you mean Table S1?

L166. Please change “analyzed” for “analysed”. In the rest of the document you use “analyse”.

L166. When you compute the ensemble mean of CMIP5 models, what resolution do you use for the ensemble mean? Which interpolation method do you use? This must be mentioned in the document.

L170. I find it inappropriate to use the expression “maximum expected quality of numerical simulations”. Please rewrite this sentence.

L187. Have the reanalysis GLORYS been used before for works in the Red Sea? If do, please add some references about it.

L238. An interesting question that shows up in this analysis is: is there a specific season when the models do a better or worst performance?

L249. You can complement the trend analysis using the Theil-Sen slope methodology which is more robust estimation.

L279. I suggest changing the structure of Figure 2 in order to facilitate its reading. Maybe you can combine bottom and top subplots in a single scatter plot with the x axis as the area and y axis as volume. In this plot each model would be a dot and the $\pm25\%$ threshold respect observed values would be a region. Models that fall inside this region are selected and the ones out of this region are discarded. Another advantage of this method is that you can show how is the performance of the 8 selected models in this criterion.

L299- I think it would be a good idea to add a new line in Figure 3 with the average of the selected models in order to better see if these models present a superior performance. This suggestion applies also for Figure SI1.

L327. Add the season name on each subplot in Figure 4. Also, it would be a good idea to add a subplot with the average of the 8 selected models for both seasons.

L345. Add titles to Figure SI2 in order to identify easily the plot corresponding to each region.

L375. Again, it would be a good idea to add a subplot in Figure 5 with the average of the 8 selected models. Please specify the units of the color palette.

L391. It would be a good idea to add a line for the average of the selected models in Figure 6. Why do you didn’t use the SST satellite data from NOAA-AVHRR taking in account that this dataset covers a longer period (1981-2017)?

L460. Please add the units of the color palette in Figure 7 and in Figure 8.

L476. It seems to me that in Figure 8 the ensemble mean of selected GCMs has the same resolution that the finest model MPI. This is not a good idea to change models with low horizontal resolution to a finer grid because in this step you are creating spurious information that was not in the data. I think is better to change the resolution to the coarser resolution and lose some information instead of creating spurious information.

L491. In order to get a better idea of the degree of these warming trends the historical period should be added to Figure 9.

L498. The second column (titled “Present”) should be removed since this column is unnecessary and does not add any information.

L516. There is an error in the word ‘projectionsis’.

L528. It is missing the closing parenthesis.

L537. I find some issues in this methodology. The first thing is that you are combining information from observations (using present day intra-seasonal variability) with GCM projections. It would be better to compare the GCM and observed MHW in the present period. After this analysis, you can use each of the selected models to construct the projections using the same methodology but using intra-seasonal present variability from selected models. At the moment, your paper does not assess the spread and uncertainty of the model projections in the MHW. This should be assessed using the projections of each of the selected models individually.

L537. Regarding the methodology to obtain the projection of daily SST there are also some issues that concern me. First, it would be good if you use a low-pass filter for obtaining the daily present anomalies (e.g. Butterlworth filter). At least, this would be good for comparing with your actual daily present anomalies. You can use a low-pass filter for SST projections in the selected models to construct your future time series. In this way you could assess the inter-model spread in the projections of the MHW.

L633. The reanalysis JRA55 is not mentioned in the Data section.

L799. I assume that right panel of the SI3 Figure is for the Outer region, but it is lacking in the figure caption.

Supplementary figures are very disorganized and shows up in the text in an aleatory order.
