# Supplementary material for: Assessment of Red Sea temperatures in CMIP5 models for present and future climate
Source: PLoS One. 2021 Jul 30;16(7):e0255505. doi: 10.1371/journal.pone.0255505 (PMC8323894; doi:10.1371/journal.pone.0255505)
Supplement: Attachment — Submitted filename: Response to Reviewers.docx [file pone.0255505.s017.docx]

**Response to editor’s comments**

Thank you for submitting your manuscript to PLOS ONE. After careful consideration, we feel that it has merit but does not fully meet PLOS ONE’s publication criteria as it currently stands. Therefore, we invite you to submit a revised version of the manuscript that fully addresses all the points raised during the review process.

We thank the editor and referee’s comments and the effort they made in reviewing our work. In the new version of the manuscript we have implemented all the points raised in the review. Thanks to those suggestions, we believe the new version of the manuscript has been significantly improved.

In particular, we have updated the figures that needed to be improved, according to the reviewer’s comments. Figures corresponding to the results of each diagnostic have been updated adding the results of the ensemble of the selected GCMs . To clarify purpose, the titles and captions of those figures have been also updated.

In order to address the lack of uncertainty in the MHWs characteristics provided for the future scenarios, we have calculated the projected changes of the MHWs characteristics for each GCMs individually. Then we use the ensemble standard deviation as a measure of the uncertainty for each diagnostic. Besides, we are aware that the supplementary figures were a bit disorganized, so we have re-ordered them to be consistent with the text.

Finally, the rest of the detailed comments have been carefully addressed to fulfill the reviewer’s requirements.

**Journal requirements**

1. Please ensure that your manuscript meets PLOS ONE's style requirements, including those for file naming. The PLOS ONE style templates can be found at

https://journals.plos.org/plosone/s/file?id=wjVg/PLOSOne_formatting_sample_main_body.pdf and

<https://journals.plos.org/plosone/s/file?id=ba62/PLOSOne_formatting_sample_title_authors_affiliations.pdf>

After a carefully revision, we believe that the manuscript meets PLOS ONE’s style requirements, including those for file naming.

2. We note that the grant information you provided in the ‘Funding Information’ and ‘Financial Disclosure’ sections do not match.

When you resubmit, please ensure that you provide the correct grant numbers for the awards you received for your study in the ‘Funding Information’ section.

The grant numbering has been checked, thanks.

3. We note that Figures 1, 4, 5, 7, 8, 10, S4 and S7 in your submission contain map/satellite images which may be copyrighted. All PLOS content is published under the Creative Commons Attribution License (CC BY 4.0), which means that the manuscript, images, and Supporting Information files will be freely available online, and any third party is permitted to access, download, copy, distribute, and use these materials in any way, even commercially, with proper attribution. For these reasons, we cannot publish previously copyrighted maps or satellite images created using proprietary data, such as Google software (Google Maps, Street View, and Earth). For more information, see our copyright guidelines: http://journals.plos.org/plosone/s/licenses-and-copyright.

 Those figures have been created by the authors, so there are no copyright issues.

4. Please include captions for ALL your Supporting Information files at the end of your manuscript, and update any in-text citations to match accordingly. Please see our Supporting Information guidelines for more information: [http://journals.plos.org/plosone/s/supporting-information](about:blank).

ok, it is done.

**Reviewer’s comments**

Revision of “Assessment of Red Sea temperatures in CMIP5 models for present and future climate”

By Agulles M and collaborators.

**General comment**

The work aims to assess the future projection of the Red Sea temperatures in the global warming scenarios of the CMIP5 project. This topic is very relevant and has been poorly studied before. In this sense, I recommend publishing this work in order to increase our knowledge of the GCMs skill to represent the mean state and variability of the temperature field in the Red Sea, as well as the possible consequences that global warming can have in this region. However, there are major comments that should be addressed before publication.

We appreciate the referee’s comments and thanks him/her for the effort he/she has made in reviewing our work. In the new version of the manuscript we have addressed all the points raised in the review.

**Major comments**

My major concerns are related to the Marine Heat Waves (MHW) methodology. The methodology used in the paper mixes the information from observations and GCMs in a way that is not usually done. I suggest using the information from the historical experiment to evaluate the GCM skills in representing the main characteristics of MHW respect to observations. As far as I know, there is daily data available for CMIP5 outputs necessary to this purpose (SSTs or air temperature at 2m in the worst case). After this, you can use the present intra-seasonal variability of each model to create the future projections. However, it is very important to keep the individual model projections in order to analyze the multi-model spread and uncertainty. In this part of your results, only the multi model mean is evaluated but in this kind of studies it is very important to discuss the differences that exist between models as a measure of uncertainty.

The reviewer is right. Our approach is not the common one as we don’t analyse directly the MHWs from the GCM outputs, but rather we assess how the changes in the mean temperature may affect the MHW. The reason is twofold:

First, at present we don’t have the capacity to download and analyse the daily outputs from the CMIP5 simulations. Furthermore, although we have proven that selected GCMs provide a reasonable modelling of the mean temperatures, it is not clear that they could be used for the intraseasonal variability in that region. So, additionally analyses should be done and at this point we would be unable to perform them. Therefore, we have discarded the possibility of analysing the GCM intraseasonal variability.

Alternatively, we focus on how the increase of the mean temperature (which we think is a robust result from the selected GCMs) may affect the MHW characteristics. This is clearly stated and justified in the manuscript (L559-L573). Moreover, this choice is not simply for practical reasons. Several studies have suggested that the most influential factor in the projected changes of MHWs will be the increase in the mean temperature and not the changes in the intraseasonal variability. This has been explained in the manuscript (L550, (Darmaraki et al., 2019; Jordà et al., 2012)). In consequence, we think that it is useful to analyse separately the role of the projected changes in the mean state from the projected changes in the intraseasonal variability.

Nevertheless, we appreciate the reviewer’s comment about the uncertainty. It is true that we didn’t address it in the manuscript while being an important issue. Therefore, we have recomputed the projected changes of MHWs for each one of the selected models. The MHWs characteristics of each model have been included in a new suplementary table (S4 Table) and the multimodel STD has been used as a measure of the uncertainty and added as a new plot (third column) in Fig 10 and S9 Fig.

**Specific comments**

I feel that the document is a little disorganized. Most of the figures do not have proper titles or the units of the color palettes, what makes difficult to read them.

Thank you for noticing this. All figures and units that the reviewer suggests changing, have been updated. The supplementary figures have been re-organized according to the explanation in the manuscript. These changes also are explained in each particular response in this document.

L47. What do you mean by “slow changes”? Do you mean changes at “long term” or changes in “low frequency variability”? Please rewrite this sentence.

Thank you. We referred to long-term changes. We have rewritten this sentence in the manuscript.

L60. There are some very interesting works about global warming and the Red Sea (e.g. “Ocean warming slows coral growth in the central Red Sea” Cantin 2010). For this reason, I suggest changing the phrase “These projections are, however, currently lacking” for something like “The assessment of these projections is limited to few works” and I also recommend citing these few works here.

The reviewer is right. We have changed the original sentence for “The assessment of these projections is limited to few works” and we have added the following references related to this:

- Shaltout, M.: Recent sea surface temperature trends and future scenarios for the Red Sea, Oceanologia, 61(4), 484–504, doi:10.1016/j.oceano.2019.05.002, 2019.
- Cantin, N. E., Cohen, A. L., Karnauskas, K. B., Tarrant, A. M., & McCorkle, D. C. (2010). Ocean warming slows coral growth in the central Red Sea. Science, 329(5989), 322–325

L123. Please include the units of the color palette (bathymetry).

This figure has been updated.

L154. I didn’t find this information in Table1. Do you mean Table S1?

Yes, we do mean that the information is in S1 Table. This has been corrected in the manuscript, thanks.

L166. Please change “analyzed” for “analysed”. In the rest of the document you use “analyse”.

This has been corrected. Thanks.

L166. When you compute the ensemble mean of CMIP5 models, what resolution do you use for the ensemble mean? Which interpolation method do you use? This must be mentioned in the document.

We have added this explanation in the methods section: “All the diagnostics have been performed into the native model grid. However, for ensemble averaging or mapping, all model fields have been bilinearly reinterpolated into a common grid. For a clearer visualization we have chosen the KAUST model grid, as it is the finest one. In order to fill the coastline gaps in GCMs (i.e. due to their coarse resolution), we have extrapolated the values from the closest GCM grid point” (L215-L221).

L170. I find it inappropriate to use the expression “maximum expected quality of numerical simulations”. Please rewrite this sentence.

Ok. We have rewritten the sentence and now it reads: “In order to have a benchmark of the state-of-the-art quality in Red Sea modelling”.

L187. Have the reanalysis GLORYS been used before for works in the Red Sea? If do, please add some references about it.

As far as we know, Glorys has only be used in a previous work of our team, entitled “Temporal evolution of temperatures in the red sea and the gulf of aden based on in situ observations” by Agulles et al. 2020. This reference has been added in the text.

L238. An interesting question that shows up in this analysis is: is there a specific season when the models do a better or worst performance?

It is an interesting question indeed. In order to address it, we have produced the following table. It shows the seasonal RMSE of the SST for each selected model (in black) and the reference simulations (in red). Some models provide better results in winter and spring (i.e. GISS-E2-R-CC, MPI-ESM-LR) respect to summer and autumn seasons, but others (i.e. IPSL-CM5A-MR or CMCC-CM) provide better results in summer and autumn. In average (last row of the table), we don’t appreciate significant differences in the ensemble as a whole. We have added a sentence in the SST seasonality section (L343).

| **SEASONAL ROOT MEAN SQUARE ERROR OF THE SST (ºC)** | | | | |
| --- | --- | --- | --- | --- |
| **Model** | **WINTER** | **SPRING** | **SUMMER** | **AUTUMN** |
| **GISS-E2-R-CC** | 0,67 | 0,70 | 0,74 | 0,78 |
| **IPSL-CM5A-MR** | 1,12 | 1,15 | 0,86 | 0,76 |
| **MPI-ESM-LR** | 0,67 | 0,54 | 0,73 | 0,88 |
| **MPI-ESM-MR** | 0,86 | 0,64 | 0,75 | 0,87 |
| **CMCC-CESM** | 0,58 | 1,02 | 1,01 | 0,45 |
| **CMCC-CM** | 1,11 | 1,34 | 0,98 | 0,54 |
| **CMCC-CMS** | 0,82 | 1,21 | 0,70 | 0,52 |
| **NORESM1-M** | 1,20 | 0,85 | 1,28 | 1,03 |
| **GLORYS** | 0,13 | 0,16 | 0,36 | 0,26 |
| **KAUST** | 0,33 | 0,27 | 0,30 | 0,25 |
| **MEAN (CMIP5)** | 0,88 | 0,93 | 0,88 | 0,73 |

L249. You can complement the trend analysis using the Theil-Sen slope methodology which is more robust estimation.

The methodology you propose could certainly work, and we have tested it. However, we don’t find significant differences with the classic linear regression we are using and the model selection would be unaltered. Thus, we have chosen to keep with the classical linear fit.

L279. I suggest changing the structure of Figure 2 in order to facilitate its reading. Maybe you can combine bottom and top subplots in a single scatter plot with the x axis as the area and y axis as volume. In this plot each model would be a dot and the $\pm25\%$ threshold respect observed values would be a region. Models that fall inside this region are selected and the ones out of this region are discarded. Another advantage of this method is that you can show how is the performance of the 8 selected models in this criterion.

We thank the reviewer for his/her suggestion. We agree that the result of this diagnostic is much more elegant changing the figure like he/she suggest. So we have updated this figure in the paper as follows:

L299- I think it would be a good idea to add a new line in Figure 3 with the average of the selected models in order to better see if these models present a superior performance. This suggestion applies also for Figure SI1.

We thank the reviewer for this suggestion. In fact, we have included the average of selected GCMs in all figures. Here we include how Figure 3 and Figure SI1 looks like now:

**Fig 3:**

**S1 Fig:**

L327. Add the season name on each subplot in Figure 4. Also, it would be a good idea to add a subplot with the average of the 8 selected models for both seasons.

Thank you for the appreciation. We have updated the figure in the paper as follows:

L345. Add titles to Figure SI2 in order to identify easily the plot corresponding to each region.

It is done, thank you. We have also included the average of the selected GCMs:

L375. Again, it would be a good idea to add a subplot in Figure 5 with the average of the 8 selected models. Please specify the units of the color palette.

Thank you. We have updated the figure as follows:

L391. It would be a good idea to add a line for the average of the selected models in Figure 6. Why do you didn’t use the SST satellite data from NOAA-AVHRR taking in account that this dataset covers a longer period (1981-2017)?

Thanks, we have updated the figure and now is:

We don’t use satellite SST for consistency with the rest of diagnostics. As explained in the manuscript, there is a very high correlation between the TEMPERSEA product and satellite SST for monthly averages (L658-L668). Thus, we have preferred to use the same product for all the diagnostics.

L460. Please add the units of the color palette in Figure 7 and in Figure 8.

Thanks for the suggestion. We have added the units in the colorbar.

L476. It seems to me that in Figure 8 the ensemble mean of selected GCMs has the same resolution that the finest model MPI. This is not a good idea to change models with low horizontal resolution to a finer grid because in this step you are creating spurious information that was not in the data. I think is better to change the resolution to the coarser resolution and lose some information instead of creating spurious information.

As we noted previously, we address all diagnostics using the original grid of the models. We only interpolate them to ease the visualization or for the ensemble averaging. Considering that model outputs in a given cell is representative of the average of what is inside that cell, it is well accepted to extrapolate the coarse resolution data towards the coastline to fill the gaps.

L491. In order to get a better idea of the degree of these warming trends the historical period should be added to Figure 9.

Thank you for the comment. We have updated this figure adding the historical period of SST (1985-2005) for the selected GCMs.

L498. The second column (titled “Present”) should be removed since this column is unnecessary and does not add any information.

Ok, it has been removed from the table. Thanks.

L516. There is an error in the word ‘projectionsis’.

Ok, it has been corrected. Thanks.

L528. It is missing the closing parenthesis.

Ok, it has been added. Thanks.

L537. I find some issues in this methodology. The first thing is that you are combining information from observations (using present day intra-seasonal variability) with GCM projections. It would be better to compare the GCM and observed MHW in the present period. After this analysis, you can use each of the selected models to construct the projections using the same methodology but using intra-seasonal present variability from selected models. At the moment, your paper does not assess the spread and uncertainty of the model projections in the MHW. This should be assessed using the projections of each of the selected models individually.

Thank you for this comment and we understand and appreciate your concern. However, as we have pointed out previously, unfortunately at present we don’t have the capacity to download and analyse the daily outputs from the CMIP5 simulations. Besides, although we have proven that selected GCMs provide a reasonable modelling of the mean temperatures, it is not clear that they could be used for the intraseasonal variability in that region. So, additionally analyses should be done and at this point we would not be able to perform them. Therefore, we have discarded the possibility of analysing the GCM intraseasonal variability.

Alternatively, we focus on how the increase of the mean temperature (which we think is a robust result from the selected GCMs) may affect the MHW characteristics. This is clearly stated and justified in the manuscript (L559-573). Moreover, this choice is not simply for practical reasons. Several studies have suggested that the most influential factor in the projected changes of MHWs will be the increase in the mean temperature and not the changes in the intraseasonal variability. This has been explained in the manuscript (L550, (Darmaraki et al., 2019; Jordà et al., 2012)). In consequence, we think that it is useful to analyse separately the role of the projected changes in the mean state from the projected changes in the intraseasonal variability.

L537. Regarding the methodology to obtain the projection of daily SST there are also some issues that concern me. First, it would be good if you use a low-pass filter for obtaining the daily present anomalies (e.g. Butterlworth filter). At least, this would be good for comparing with your actual daily present anomalies. You can use a low-pass filter for SST projections in the selected models to construct your future time series. In this way you could assess the inter-model spread in the projections of the MHW.

We are sorry but we are not sure of understanding the reviewer’s concerns, so we apologize if our answer doesn’t go in the right direction.

We agree that using a low-pass filter instead of removing the monthly mean is more accurate. However, in practice the differences between both methods are very small. Regarding applying the filter to GCM outputs, as we mentioned before we don’t have the daily GCM outputs, so it is pointless to apply the low-pass filter to GCMs data. Finally, about the inter-model spread, we have somehow addressed this point as explained previously. In particular, we assess what is the impact of the mean temperature change from each GCM separately, and then compute the ensemble average and ensemble spread as a measure of the uncertainty.

L633. The reanalysis JRA55 is not mentioned in the Data section.

The reviewer is right, so we have included an “Atmospheric Reanalysis” subsection in the Data and Methods section (L146).

L799. I assume that right panel of the SI3 Figure is for the Outer region, but it is lacking in the figure caption.

We are sorry for the misunderstanding. The right panel does not correspond to the Outer Region, but it represents the 3d temperature anomalies projected in the Red Sea basin for the selected GCMs. To clarify this, we have added titles in the panels and updated the legend.

Supplementary figures are very disorganized and shows up in the text in an aleatory order.

We have carefully revised all figures names and all supplementary figures have been properly organized. Thanks for the comment.
